# The investigation of resting-state functional connectivity in male sprinters and endurance runners brains based on fNIRS

**DOI:** 10.1038/s41598-025-99573-w

**Published:** 2025-04-29

**Authors:** Mengqi Liu, Wenyan Zhao, Xiaocong Yan, Zhenghao Xue, Ying Qin

**Affiliations:** 1 Experimental Center for Development and Evaluation of Sports Ability, Graduate School, Harbin Institute of Physical Education, Harbin, Heilongjiang Province China; 2 Harbin Institute of Physical Education, College of Sports and Human Sciences, Harbin, Heilongjiang Province China; 3 Experimental Center for Development and Evaluation of Motor Ability, Harbin Institute of Physical Education, Institute of Sports Science, Harbin, Heilongjiang Province China

**Keywords:** Aerobic exercise, Anaerobic exercise, Resting-state functional connectivity, Functional near-infrared spectroscopy, Neuroscience, Physiology, Neurology

## Abstract

Objective To investigate the impact of extended aerobic and anaerobic exercise on cerebral activity by analyzing the functional connectivity strength attributes of the cerebral cortex in endurance runners (aerobic) and sprint athletes (anaerobic) during the resting state with fNIRS. Method Thirteen sprinters and twelve endurance runners were assessed using a functional near-infrared spectroscopic imaging system to quantify resting-state functional connection strengths for HbO2, HbR, and HbT across the brain, namely in the prefrontal and primary motor cortex. Results (1) In the examination of functional connectivity of HbO2, the overall functional connection strength of the anaerobic group exceeded that of the aerobic group. In the regions of interest, the functional connection strength in the left and right prefrontal cortex of the anaerobic group surpassed that of the aerobic group. However, the functional connectivity strength in the right primary motor cortex of the aerobic group was greater than that of the anaerobic group. In comparisons between regions of interest, the functional connection strength between the left and right prefrontal cortex was greater in the anaerobic group. In contrast, the aerobic group had a more pronounced functional connectivity strength between the left and right primary motor cortex. (2) The functional connectivity study of HbR indicated that the mean whole-brain functional connection strength in the anaerobic group surpassed that of the aerobic group; however, no significant differences were seen between the two groups in intra- and inter-ROI comparisons. (3) The functional connectivity study of HbT indicated that the average brain-wide functional connection strength in the aerobic group surpassed that of the anaerobic group. In the regions of interest, the anaerobic group had greater functional connectivity strength in the right prefrontal cortex. In contrast, the aerobic group demonstrated more pronounced functional connectivity strength in the right primary motor cortex. The aerobic group exhibited greater functional connection strength between M1-R and M1-L throughout the regions of interest. Conclusion The aerobic group had enhanced functional brain connectivity in the primary motor cortex, whereas the anaerobic group demonstrated superior functional brain connectivity in the prefrontal lobe. Various exercise modalities will have distinct influences on neuroplasticity across different brain regions, establishing a novel theoretical framework for exercise training and clinical rehabilitation.

## Introduction

Physical activity provides significant benefits in improving physical fitness and fostering cognitive wellness. Prolonged engagement in physical activity can markedly improve cognitive performance and decelerate the brain’s ageing process^1^. Moreover, engagement in physical activity helps mitigate sadness and anxiety while enhancing an individual’s emotional regulation and adaptability to stress. Exercise specificity profoundly influences physiological and psychological systems; low-intensity sustained aerobic exercise and high-intensity intermittent anaerobic exercise induce adaptive modifications in the cardiovascular system^3^(cardiac output and stroke volume), the musculoskeletal system^4^(muscle fiber hypertrophy, mitochondrial density, and motor unit recruitment), and the peripheral nervous system via distinct metabolic pathways. Advancements in neuroimaging have directed researchers’ attention to the impact of various movement patterns on the central nervous system, notably as seen by the intensity of resting-state functional connectivity. Functional connectivity strength denotes the synchronized activities or relationships among various brain regions utilized to comprehend the brain’s functions across diverse states or tasks by quantifying the temporal correlation of neural signals between distinct brain areas^5,6^. Increased functional connection strength enhances the efficiency of information flow, integration, and collaboration among brain regions. Resting State Functional Connectivity (RSFC) is a metric specifically formulated to assess the interconnection of various brain regions or tissues during the resting state. The resting state does not represent complete ‘rest’; instead, it reflects ‘internal brain activity’ or ‘spontaneous brain activity.’ The brain’s energy consumption at rest is nearly equivalent to that during task performance^8^, indicating that the intensity of functional connection can characterize resting-state brain activity. Prior research has concentrated on the impact of individual exercise sessions on the strength of functional connectivity within the cerebral cortex^9–11^. At the same time, fewer investigations have explored the effects of prolonged aerobic and anaerobic training on resting-state functional connectivity in the brain, particularly among athletes. Functional near-infrared spectroscopy (fNIRS) is a noninvasive, real-time technique for monitoring cerebral activity that is safe, portable, quiet, and relatively cost-effective. It is less cumbersome than other neurofunctional imaging modalities, such as functional magnetic resonance imaging (fMRI), electroencephalography (EEG), and positron emission tomography (PET). The fNIRS technique for scanning cortical activity during the brain’s resting state accurately captures the cumulative impacts of prolonged experience, as it is unaffected by external variables^12^. Prolonged systematic training results in distinct patterns of neuronal activation throughout several pertinent brain regions within the cerebral cortex network, potentially correlating with the motor talents or skills necessary for diverse movement patterns. This study identified the primary motor cortex (M1) and prefrontal cortex (PFC) as regions of interest (ROIs), which primarily govern motor control and cognitive functions, respectively, exhibiting significant adaptability in both structure and function based on training experience^12,13^. Anaerobic activity, such as sprinting, necessitates rapid decision-making and significant cognitive regulation, and the robustness of functional connections in the prefrontal cortex (PFC), a center for advanced cognition, may be enhanced with high-repetition explosive training^14^. Aerobic activity, such as endurance running, facilitates synaptic remodeling and long-term potentiation (LTP) in the primary motor cortex (M1) by elevating brain-derived neurotrophic factor (BDNF) levels^15^. Given the distinct impact of various exercise modalities on cerebral function, we hypothesized that resting-state prefrontal cortex functional connectivity strength would be significantly greater in chronically anaerobically trained athletes compared to aerobically trained athletes, whereas M1 functional connectivity strength would be superior in aerobically trained athletes. This study aims to compare the functional connectivity characteristics of various brain regions in the resting state between athletes engaged in long-term aerobic and anaerobic training utilizing the FNIRS technique. The objective is to offer novel research insights into how prolonged aerobic and anaerobic training fosters neural plasticity in the brain through a contemporary neuroimaging approach and to clarify the impact of distinct exercise modalities on the functional connectivity of brain networks, thereby enhancing the understanding of the neurological mechanisms underlying exercise training in the promotion of brain health. We will clarify the impact of various exercise modalities on the functional connectivity of brain networks and enhance the understanding of the neural mechanisms behind exercise training for brain health.

## Results

This study encompasses measurements of HbO_2_, HbR, and HbT. Hemodynamic changes in cerebral activity can be deduced by analyzing the differential absorption of light by oxyhemoglobin (HbO_2_) and deoxyhemoglobin (HbR), which absorb different wavelengths. HbT denotes the total of HbO_2_ and HbR, reflecting fluctuations in both HbO_2_ and HbR and providing comprehensive insights on blood supply and utilization in cerebral regions. This study discovered four intra-ROI connectivity patterns: PFC-L-PFC-L, PFC-R-PFC-R, M1-R-M1-R, and M1-L-M1-L. The six inter-ROI connectivity patterns included PFC-L-PFC-R, PFC-L-M1-R, PFC-L-M1-L, PFC-R-M1-R, PFC-R-M1-R, PFC-R-M1-L, and M1-R-M1-L.

### Comparison of functional connection strength in the brain among HbO_2_-based groups

**Fig. 1:**
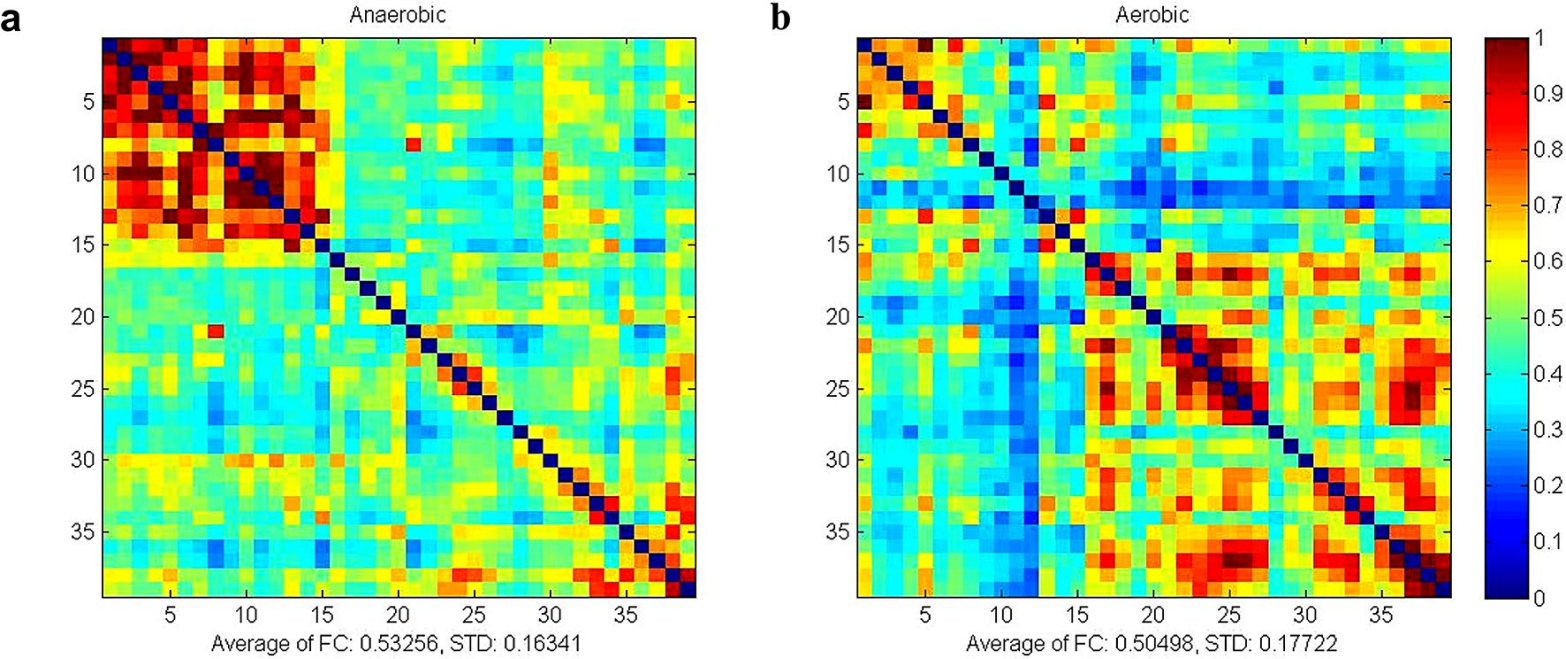
Mean intensity matrix of HbO2 resting-state functional connectivity in both groups of athletes (Red color indicates high connection strength, blue color indicates low connection strength). **a**: matrix of mean intensity of functional connectivity in the anaerobic group. **b**: matrix of mean intensity of functional connectivity in the aerobic group.

**Fig. 2:**
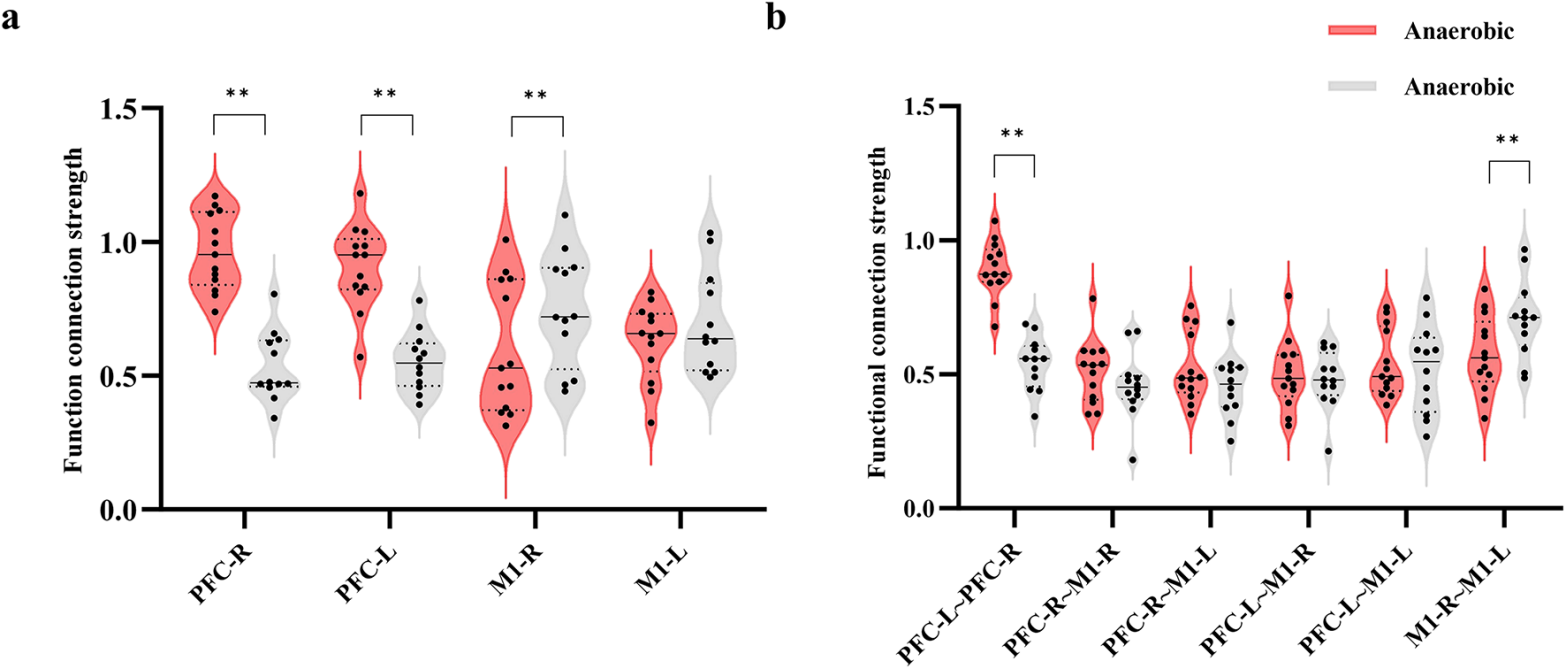
Comparison of functional connectivity strength between HbO2-based brain compartments. **a**: Strength of functional connectivity between the same brain regions. **b**: Strength of functional connectivity between different brain regions. **: *P* < 0.01.

Figure 1 illustrates the average strength of whole brain HbO_2_ resting state functional connectivity between aerobic and anaerobic groups. The mean functional connectivity strength in the anaerobic group was (0.53 ± 0.16), while in the aerobic group it was (0.52 ± 0.17). The average strength of whole-brain functional connectivity in the anaerobic group exceeded that of the aerobic group. Comparative analyses of functional connectivity strength within the four regions of interest (ROIs) and among the six ROI pairs in the two athlete groups demonstrated highly significant differences in correlation coefficients for PFC-L (*P* = 0.003, Hedges’ g = 1.48, 95% CI[0.74, 2.22]), PFC-R (*P* = 0.003, Hedges’ g = 1.49, 95% CI[0.75, 2.23]), M1-R (*P* = 0.003, Hedges’ g=−1.34, 95% CI[−2.07, −0.61]), PFC-L-PFC-R (*P* = 0.004, Hedges’ g = 1.50, 95% CI[0.76, 2.24]), and M1-R-M1-L (*P* = 0.001, Hedges’ g=−1.77, 95% CI[−2.26, −0.98]). The functional connectivity strength between PFC-L regions was greater in the anaerobic group (*r* = 0.884 ± 0.186) compared to the aerobic group (*r* = 0.608 ± 0.198), and the connectivity between PFC-R regions was also higher in the anaerobic group (*r* = 0.928 ± 0.226) than in the aerobic group (*r* = 0.590 ± 0.227). The functional connectivity between the PFC-L and PFC-R areas was greater in the anaerobic group (*r* = 0.854 ± 0.180) compared to the aerobic group (*r* = 0.576 ± 0.196). The functional connection strength between the M1-R areas was greater in the aerobic group (*r* = 0. The functional connectivity in the aerobic group (*r* = 0.802 ± 0.160) was superior to that in the anaerobic group (*r* = 0.519 ± 0.154) within the M1-R-M1-L areas, indicating a greater amount of connectivity between them. The results are shown in Fig. 2.

### Comparison of brain functional connectivity strength between HbR-based groups

**Fig. 3:**
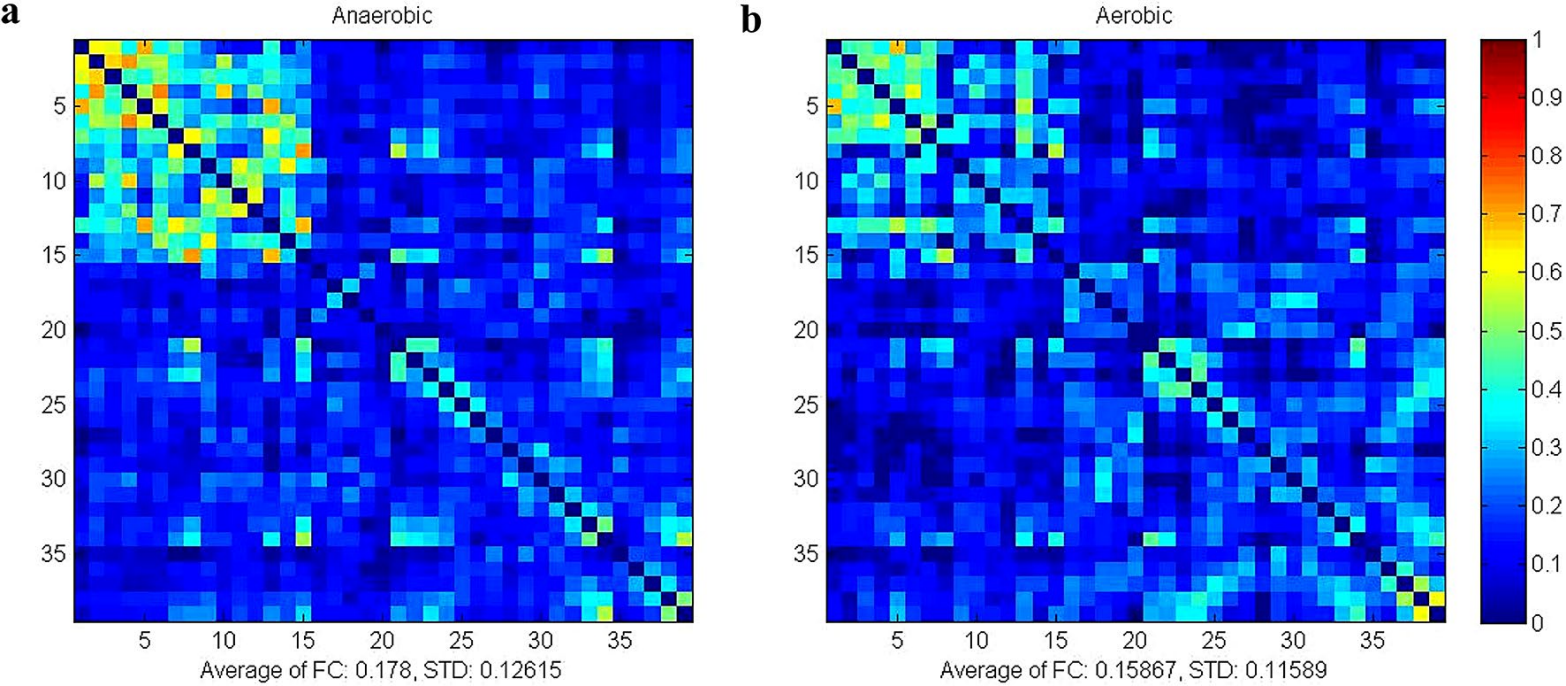
Mean intensity matrix of HbR resting-state functional connectivity in both groups of athletes. **a**: matrix of mean intensity of functional connectivity in the anaerobic group. **b**: matrix of mean intensity of functional connectivity in the aerobic group.

**Fig. 4:**
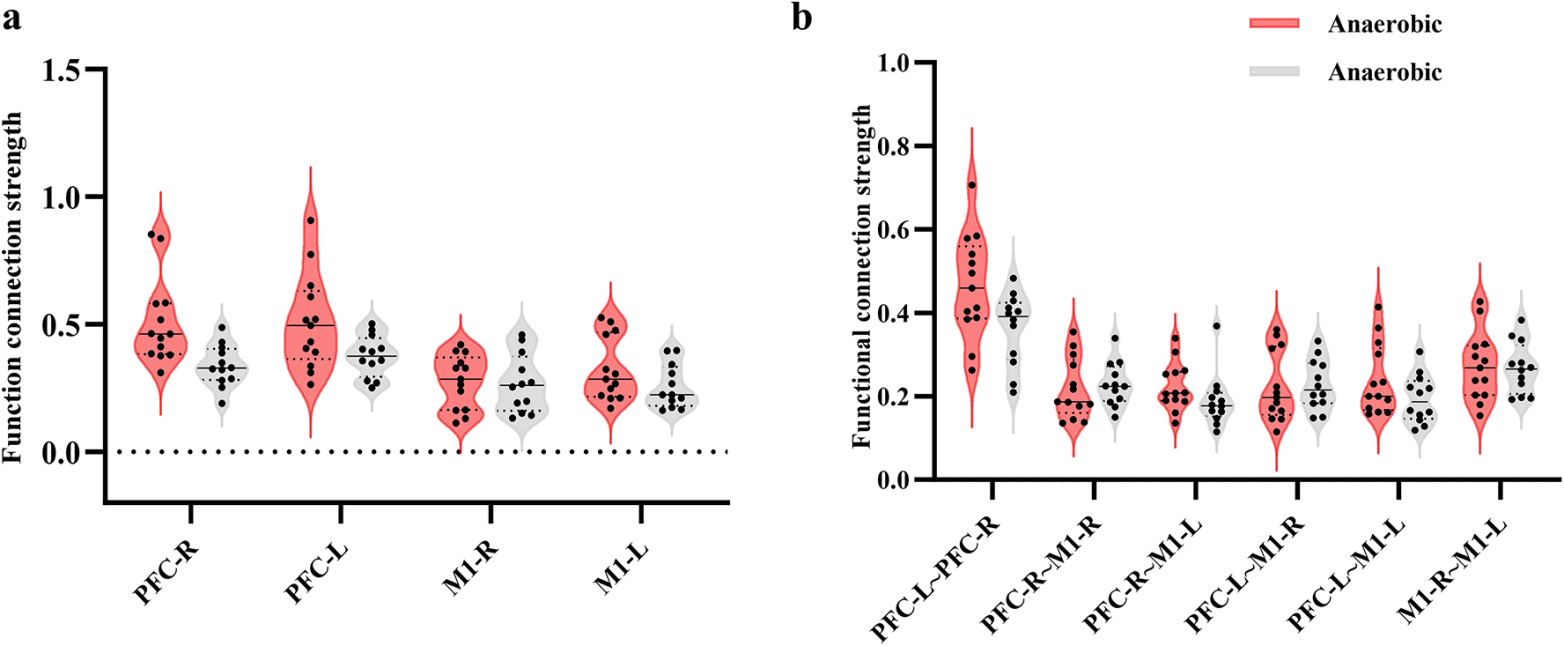
Comparison of functional connectivity strength between HbR-based brain regions**a**: Strength of functional connectivity between the same brain regions**b**: Strength of functional connectivity between different brain regions.

Figure 3 illustrates the average strength of whole-brain HbR resting-state functional connectivity between the aerobic and anaerobic groups. The mean strength of whole-brain functional connectivity in the anaerobic group was 0.18 ± 0.13, while in the aerobic group, it was 0.17 ± 0.12, indicating that the anaerobic group’s functional connectivity strength was superior to that of the aerobic group. Comparative analyses of the mean degree of functional connectivity within and among the regions of interest (ROIs) indicated that the disparity between the two groups based on hemoglobin redox state (HbR) was not statistically significant (*P* > 0.05). The results are shown in Fig. 4.

### Comparison of brain functional connectivity strength between HbT-based groups

**Fig. 5:**
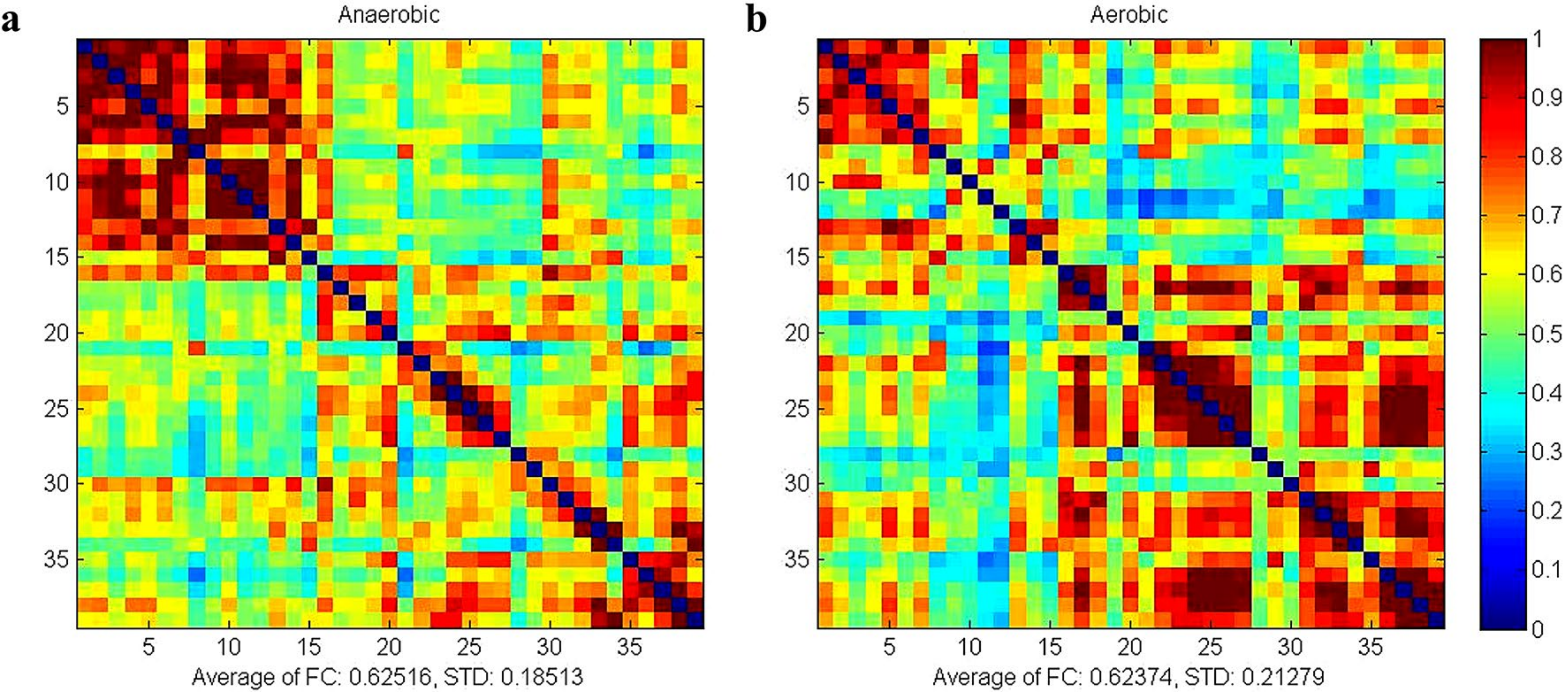
Mean intensity matrix of HbT resting-state functional connectivity in both groups of athletes**a**: matrix of mean intensity of functional connectivity in the anaerobic group**b**: matrix of mean intensity of functional connectivity in the aerobic group.

**Fig. 6:**
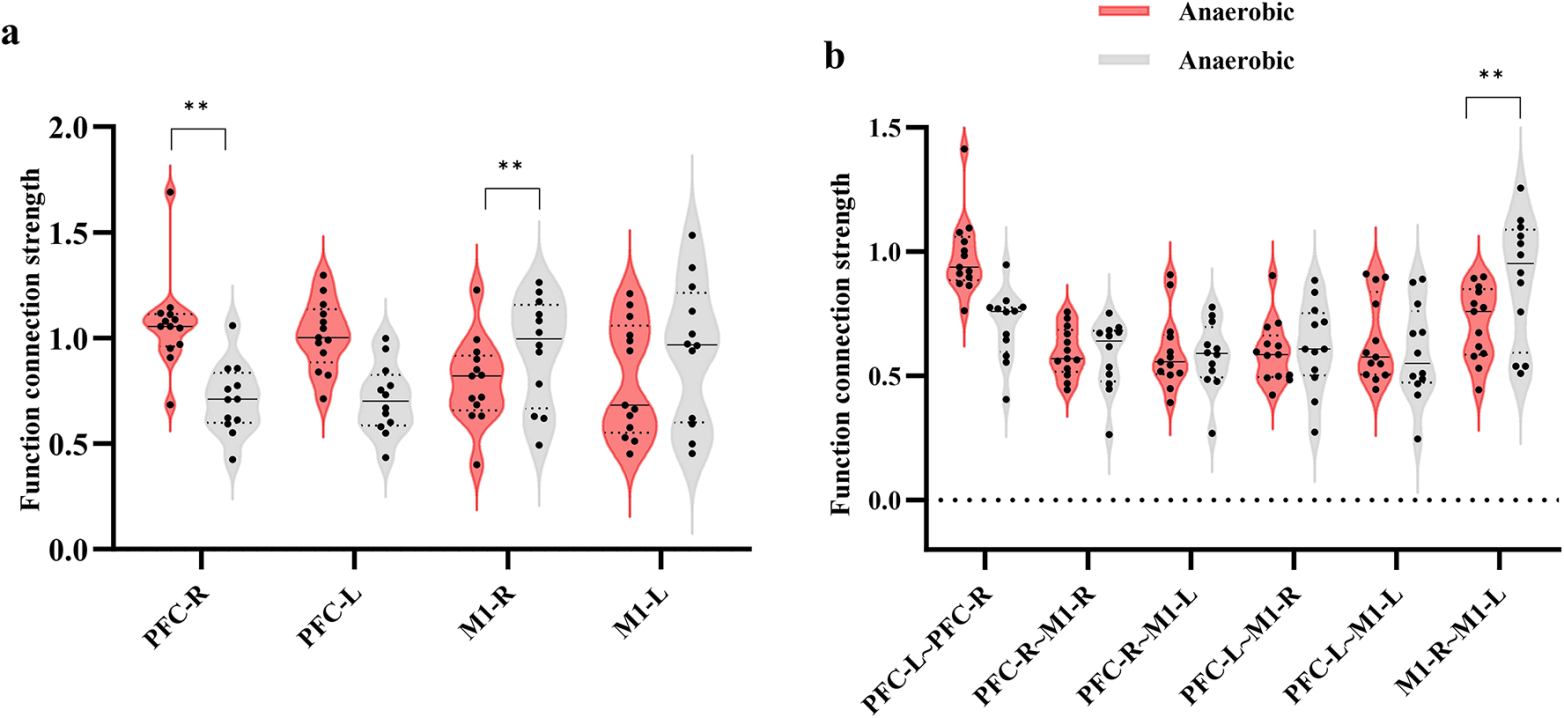
Comparison of functional connectivity strength between HbT-based brain regions. **a**: Strength of functional connectivity between the same brain regions. **b**: Strength of functional connectivity between different brain regions. **: *P* < 0.01.

Figure 5 illustrates the average strength of whole brain HbT resting state functional connectivity between aerobic and anaerobic groups. The anaerobic group exhibited an average whole-brain functional connectivity of (0.63 ± 0.19). In contrast, the aerobic group demonstrated a higher average of (0.65 ± 0.21), indicating superior functional connectivity strength in the aerobic group compared to the anaerobic group. Comparative analyses of the strength of functional connectivity within the four regions of interest (ROIs) and among the six ROI pairs in the two athlete groups revealed significant differences in correlation coefficients for the PFC-R (*P* = 0.03, Hedges’ g = 1.05, 95% CI[0.35, 1.75]), M1-R (*P* = 0.02, Hedges’ g=−1.27, 95% CI[−1.99, −0.55]), and M1-R-M1-L (*P* = 0.01, Hedges’ g=−1.40, 95% CI[−2.14, −0.66]) regions. The functional connectivity strength was greater in the aerobic group (*r* = 1.100 ± 0.294) compared to the anaerobic group (*r* = 0.720 ± 0.305) in the M1-R region, and it was also higher in the aerobic group (*r* = 0.992 ± 0.305) than in the anaerobic group between the M1-R and M1-L regions. The functional connection strength in the aerobic group (*r* = 0.992 ± 0.305) surpassed that of the anaerobic group (*r* = 0.641 ± 0.148). The results are shown in Fig. 6.

## Discussion

The prefrontal cortex is essential for coordinating and executing motions, as it is involved in goal planning, decision making, motivation, and cognitive control^13^. The resting state is defined as the intrinsic activity of neurons in the brain during periods devoid of specialized cognitive tasks, illustrating the essential characteristics of neural function. The level of functional connection during the resting state offers a crucial viewpoint for evaluating the impact of aerobic and anaerobic exercise on cerebral function. The findings indicated that the strength of HbO_2_functional connection among the PFC-L, PFC-R, and PFC-L-PFC-R areas was greater in the anaerobic group compared to the aerobic group, and the connectivity strength between PFC-R surpassed that of PFC-L. The intensity of HbT functional connectivity between PFC-R areas surpassed that of the aerobic group. There is a paucity of prior research on aerobic and anaerobic activity at rest; nonetheless, this aligns with earlier findings during exercise^16^; Kojima S et al. discovered that prefrontal functional connection strength was markedly greater in the aerobic group compared to the anaerobic group. Kojima S et al. observed a notable elevation in prefrontal cortex HbO_2_following the aerobic threshold (AT) during incremental loading exercise, with the oxygenation rate in the right prefrontal lobe preceding that of the left prefrontal lobe. Brief intervals of anaerobic exercise lead to increased prefrontal oxygenated hemoglobin levels^16–18^, thereby reducing deoxyhemoglobin, a phenomenon that may intensify with extended anaerobic training.

Such changes may arise from external settings or learning demands that modify brain structure or function, a phenomenon referred to as neuroplasticity^19^. Research indicates that prolonged training substantially influences neuronal alterations, cerebral architecture, and neural network interconnectivity^20^. Sprinting is a quintessential anaerobic workout primarily fuelled by the phosphagen system, characterized by brief duration, high intensity, and rapid energy expenditure. Anaerobic exercise elicits significant alterations in neuronal function and brain structure^22^. Prior research indicates that anaerobic exercise generates lactic acid, which prompts the release of vascular endothelial growth factor (VEGF), resulting in heightened capillary density in the brain, and this augmentation in capillary density may modify the interactions between neurons and blood vessels^23^. Lactate additionally facilitates neurogenesis in the adult hippocampus^24^, and the proper functioning of the hippocampus is a crucial prerequisite for the prefrontal lobe to execute cognitive activities successfully^25^, hence allowing individuals to process information efficiently. Besides neurovascular adaptations, anaerobic exercise may induce morphological alterations in the brain, resulting in an increase in grey matter volume in the basal ganglia of athletes who have participated in extended anaerobic exercise^22^, which is closely associated with the regulation of motor skills. The basal ganglia predominantly receive signals from the prefrontal brain, which may represent the initial motor intention^26^. The elevated strength of functional connection in the prefrontal lobe within the anaerobic group may be attributed to neural adaptation, structural alterations in the brain, and additional factors. Modern sprinting tactics depend on the synchronization of the neuromuscular system to attain rapid movement, and proficient execution of these techniques enhances the athlete’s focus, cognitive capacity, and overall performance throughout both the acceleration and sprint stages of the race^27^. The quality of strength is fundamental for athletes to execute technical motions; for sprinters, explosive power and speed endurance are significantly enhanced through resistance training. Research indicates that resistance training improves working memory and executive function^28,29^, potentially linked to increased levels of cognition-related hormones such as brain-derived neurotrophic factor (BDNF), irisin, and insulin-like growth factor-1 (IGF-1)^13^. Resistance training induces increased hormone levels that enhance neuroplasticity and cognitive function^30^. The modified brain function in the anaerobic group may be ascribed to the characteristics of extended engagement in sport-specific skill training and energy sources that improve prefrontal function.

The primary motor cortex is the key area of the cerebral cortex responsible for planning, controlling, and executing voluntary movements. The findings indicated that the strength of HbO_2_ functional connectivity between the M1-R and M1-R-M1-L regions was significantly greater in the aerobic group than in the anaerobic group, and the strength of HbT functional connectivity within the M1-R region was likewise superior in the aerobic group compared to the anaerobic group. Prior research indicated that comparisons of the strength of resting-state functional connectivity of HbO_2_in the motor cortex, conducted before and after moderate-intensity aerobic exercise, demonstrated significant differences solely within the high fitness level group among college students of varying fitness levels^5^. This study compared the resting-state functional connectivity strength of HbO_2_in the motor cortex with that of the high-fitness level group. This indicates that fitness level may diminish the impact of aerobic exercise on functional brain connections, implying that low-intensity aerobic exercise could be more advantageous for individuals with elevated fitness levels. The theory of transitory frontal lobe hypoplasia^30^indicates that extended physical activity results in a redistribution of the brain’s finite metabolic resources and that exercise stimulates neural activation associated with motor patterns, sensory input processing, and autonomic regulation, consequently diminishing cortical activity in regions not pertinent to exercise^33,34^. Extended aerobic exercise generates structural and functional alterations in the brain, with the motor cortex significantly influencing aerobic activity^35–37^, potentially augmenting the strength of M1 connections. Endurance running is a conventional aerobic activity defined by aerobic oxidative energy production and sustained availability of energy substrates. The disparity in M1 between the aerobic and anaerobic groups may stem from many physiological factors. Aerobic exercise elevates BDNF levels at the cellular level, which is essential for neuronal remodeling, regulation of synaptic plasticity, and neurotransmitter release^34^. Endurance runners exhibit augmented grey matter volume and cortical surface area in the left precentral gyrus, along with improved functional connectivity between the right postcentral gyrus and precentral gyrus^38,39^. These regions are essential for conveying motor impulses to the body’s muscles and are intricately linked to motor control. Research demonstrated that neoangiogenesis and capillary diameter increased in the rat motor cortex following a 5-week aerobic exercise regimen^38,39^. Aerobic exercise influences the function and organization of the primary motor cortex via many mechanisms that improve motor control and execution. This study clarifies the relationship between aerobic exercise and the primary motor cortex.

### Limitations

This study has certain drawbacks. The limited sample size may restrict the generalisability of the findings; nonetheless, post hoc efficacy studies validated the statistical power required to identify bigger effect sizes. Secondly, while we accounted for acute factors (e.g., alcohol intake, late nights, etc.) during the 72-hour interval, enduring lifestyle disparities (e.g., food, living conditions, etc.) between aerobic and anaerobic athletes may remain and influence the study’s outcomes. Notwithstanding these constraints, our results offer innovative perspectives on exercise-induced neurovascular changes and underscore the utility of functional near-infrared spectroscopy (fNIRS) in athlete populations. Subsequent research should corroborate these findings by analyzing diverse viewpoints and bigger sample sizes across gender, age, and sport.

## Conclusion

This study examined the level of functional connection in various brain regions between sprinters and endurance runners during the resting state. Sprinters had greater functional connectivity strength in the prefrontal lobe, whereas endurance runners demonstrated enhanced functional connectivity strength in the motor cortex. The study’s findings possess significant practical implications; specifically, the ratio of aerobic to anaerobic training was modified based on varying exercise requirements to facilitate the synergistic improvement of cognitive and motor performance. The study’s results have implications for clinical rehabilitation, indicating that aerobic exercise facilitates the recovery of motor area function. In contrast, anaerobic exercise enhances prefrontal connectivity, thereby improving executive function and emotional regulation in patients with cognitive or emotional disorders.

## Materials and methods

### Participants

A total of 25 subjects were recruited from Harbin Sports Institute for this study and were divided into 2 groups. Thirteen sprinters with many years of anaerobic training (100, 200, 400 m, 110 m hurdles) were assigned to the anaerobic group. At the same time, twelve endurance runners who have been trained aerobically for many years (5,000 and 10,000 m run, 10 km race walk) were assigned to the aerobic group. There was no significant difference between the two groups of subjects in terms of age, height, and years of training (*P* > 0.05), as shown in Table 1. Inclusion Criteria: (1) Normal intelligence, no history of mental illness or brain injury; (2) Right-handedness was confirmed using the Edinburgh Habitual Hand Scale; (3) No late night, alcohol consumption, or strenuous exercise in the 3 days prior to the test; (4) Athletes in both anaerobic and aerobic groups were at the level of national level 2 athletes or above. Exclusion Criteria: (1) broken skin or disease on the head; (2) inability to concentrate on the experiment. The design and conduct of this study were approved by the Ethics Committee of Harbin Institute of Physical Education (2024019) and strictly followed the guidelines of the Declaration of Helsinki. To ensure the ethical nature of the study and to protect the rights and interests of the subjects, subjects signed a written informed consent form before the experiment.

**Table 1:** Basic information of subjects.

Group	*n*	Age(years)	height(cm)	training years
Anaerobic	13	21. 15 ± 0.25	179.5 ± 3.40	7.62 ± 0.40
Aerobic	12	21.50 ± 0.35	21.50 ± 0.35	7.50 ± 0. 12
*P*	-	0.73	0.72	0.78

### Experimental instruments

This investigation utilized a NirSmart-3000 A device (Danyang Huichuang Medical Equipment Co., Ltd., China) to measure constantly and document fluctuations in the concentrations of cerebral oxyhemoglobin (HbO_2_), deoxyhemoglobin (HbR) and total hemoglobin (HbT) throughout the job. The system comprises a near-infrared light-emitting diode (LED) and an avalanche photodiode (APD) as the detector, operating at wavelengths of 730 nm and 850 nm, respectively, with a sampling rate of 11 Hz.

### Experimental procedure

**Fig. 7:**
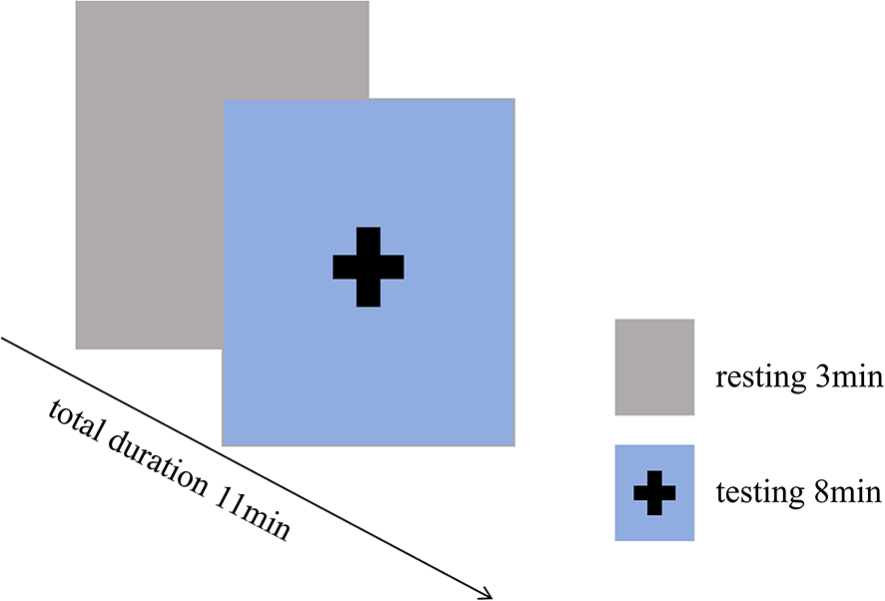
FNIRS test paradigm.

Prior to the experiment, the subjects engaged in no warm-up or preparatory activities; they remained seated in the test room, resting quietly for 3 min. Subsequently, they donned a head cap, closed their eyes, and maintained wakefulness while refraining from habitual thought processes, limb movement, and head bobbing, during which resting state data was collected for 8 min. A specimen of the examination is illustrated in Fig. 7.

### Probe arrangement

**Fig. 8:**
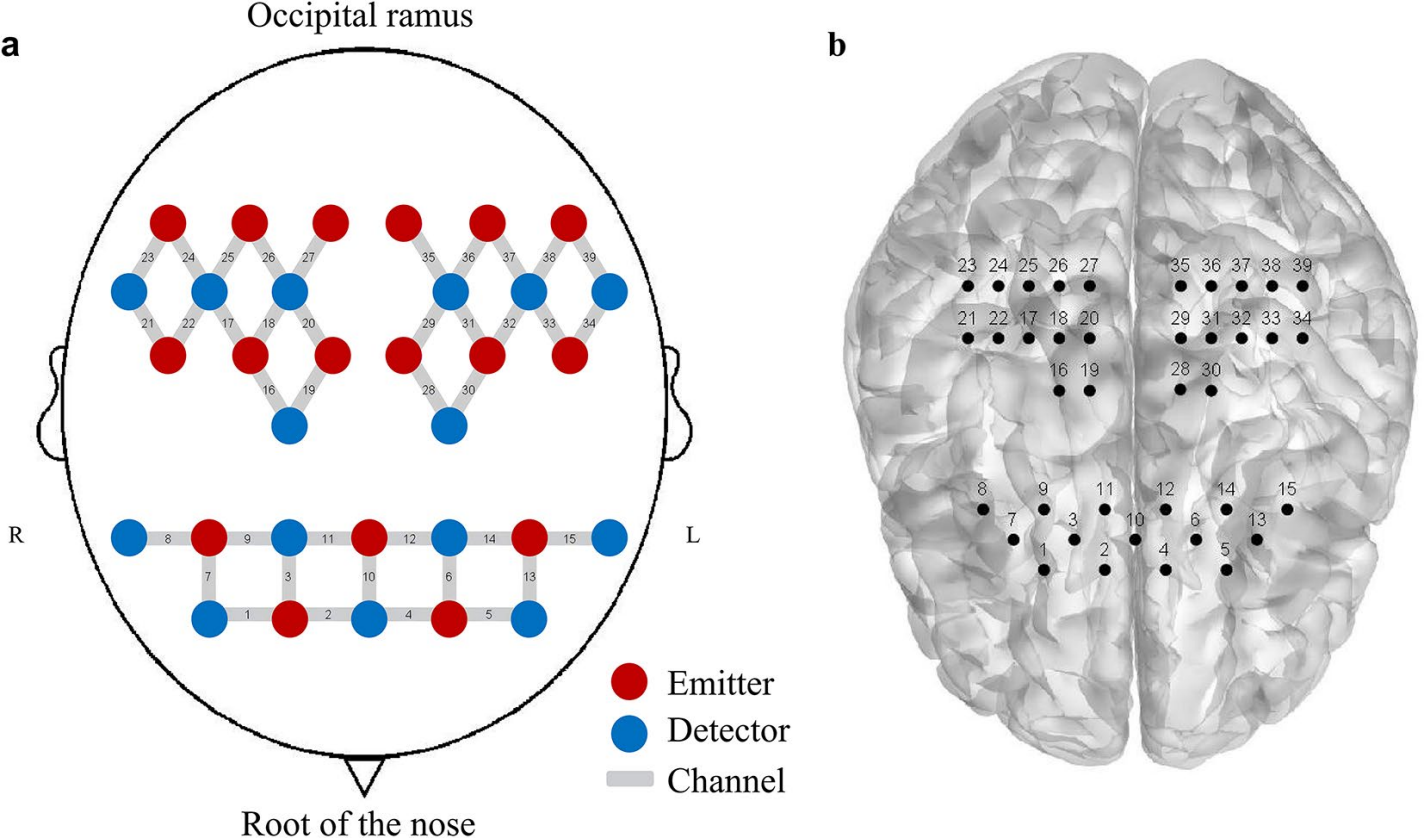
Functional near-infrared spectrogram. **a**: Probe arrangement of 17 emitters (red dots) and 15 detectors (blue dots) measuring prefrontal and primary motor cortex. **b**: Anatomical location of each channel (NirSmart-3000 A, http://www.hcmedx.cn).

The configuration of the probes is seen in Fig. 8. The position of the nasal root at the junction of the occipital ramus and the anterior points of the right and left ears was identified as the Cz placement site in accordance with the International 10–20 method. This experiment utilized 17 transmitting probes and 15 receiving probes to establish 39 active channels, with an average inter-probe distance of 3 cm (ranging from 2.7 to 3.3 cm). The selection of regions of interest (ROIs) in this investigation was based on the Brodmann partitioning system, which delineates brain areas. The chosen ROIs included the prefrontal cortex (PFC) and primary motor cortex (M1), with the associated channels for each ROI presented in Table 2.

**Table 2:** Interest area and corresponding channel arrangement.

ROIs	Cerebral hemisphere	channel arrangement
prefrontal	right	CH1,CH2,CH3,CH7,CH8,CH9,CH10,CH11
	left	CH4,CH5,CH6,CH12,CH13,CH14,CH15
Primary motor cortex	right	CH16,CH17,CH18,CH19,CH20,CH21,CH22, CH23,CH24,CH25,CH26,CH27
	left	CH28,CH29,CH30,CH31,CH32,CH33,CH34, CH35,CH36,CH37,CH38,CH39

### Probe arrangement

The data was processed and analyzed through the NirSpark toolkit. The specific steps are as follows: 1. Quality testing: the raw data are processed by signal quality testing tools;2. Data clipping: eliminating time series that are not relevant to the experimental data;3. Motion correction: The motion artifact signal is modeled using spline interpolation and then removed from the test signal;4. Filtering: High-pass filtering (0.01 Hz) and low-pass filtering (0.2 Hz) are set using band-pass filtering for noise reduction and baseline drift correction;5. Signal conversion: The measured optical density signal is transformed into a signal of blood oxygen concentration change by the Beer-Lambert law.6. Functional connectivity strength calculation: The Pearson correlation coefficient between two channels was calculated, and the resting-state functional connectivity strength of 39 channels was calculated by Fisher r-to z conversion.

### Statistical analysis

Statistical analyses were conducted utilizing GraphPad Prism 10.0 software. The normality of functional connectivity strength data was assessed via the Shapiro-Wilk test and validated for homogeneity of variance using the Levene test, presented as (mean ± SD). The independent samples t-test (*p* < 0.05) was employed to evaluate differences in functional connectivity strength between groups. Numerous hypothesis tests among areas of interest (ROIs) were adjusted utilizing the false discovery rate (FDR) approach, and all statistics from this experiment underwent FDR correction. To evaluate the practical significance of intergroup differences, effect sizes were calculated as Hedges’ g with 95% confidence intervals, and sample sizes were examined through post hoc efficacy analyses using G*Power 3.1, demonstrating a statistical power of 89% for identifying large effect sizes (g > 1.3).

## Data Availability

The datasets generated during and/or analysed during the current study are available from the corresponding author on reasonable request.
